# Host-mediated microbiome engineering (HMME) of drought tolerance in the wheat rhizosphere

**DOI:** 10.1371/journal.pone.0225933

**Published:** 2019-12-04

**Authors:** Michael D. Jochum, Kelsey L. McWilliams, Elizabeth A. Pierson, Young-Ki Jo

**Affiliations:** 1 Department of Plant Pathology & Microbiology, Texas A&M University, College Station, Texas, United States of America; 2 Department of Horticultural Sciences, Texas A&M University, College Station, Texas, United States of America; University of North Carolina at Chapel Hill, UNITED STATES

## Abstract

Host-mediated microbiome engineering (HMME) is a strategy that utilizes the host phenotype to indirectly select microbiomes though cyclic differentiation and propagation. In this experiment, the host phenotype of delayed onset of seedling water deficit stress symptoms was used to infer beneficial microbiome-host interactions over multiple generations. By utilizing a host-centric selection approach, microbiota are selected at a community level, therein using artificial selection to alter microbiomes through both ecological and evolutionary processes. After six rounds of artificial selection using host-mediated microbiome engineering (HMME), a microbial community was selected that mediated a 5-day delay in the onset of drought symptoms in wheat seedlings. Seedlings grown in potting medium inoculated with the engineered rhizosphere from the 6^th^ round of HMME produced significantly more biomass and root system length, dry weight, and surface area than plants grown in medium similarly mixed with autoclaved inoculum (negative control). The effect on plant water stress tolerance conferred by the inoculum was transferable at subsequent 10-fold and 100-fold dilutions in fresh non-autoclaved medium but was lost at 1000-fold dilution and was completely abolished by autoclaving, indicating the plant phenotype is mediated by microbial population dynamics. The results from 16S rRNA amplicon sequencing of the rhizosphere microbiomes at rounds 0, 3, and 6 revealed taxonomic increases in proteobacteria at the phylum level and betaproteobacteria at the class level. There were significant decreases in alpha diversity in round 6, divergence in speciation with beta diversity between round 0 and 6, and changes in overall community composition. This study demonstrates the potential of using the host as a selective marker to engineer microbiomes that mediate changes in the rhizosphere environment that improve plant adaptation to drought stress.

## Introduction

**Fig 1:**
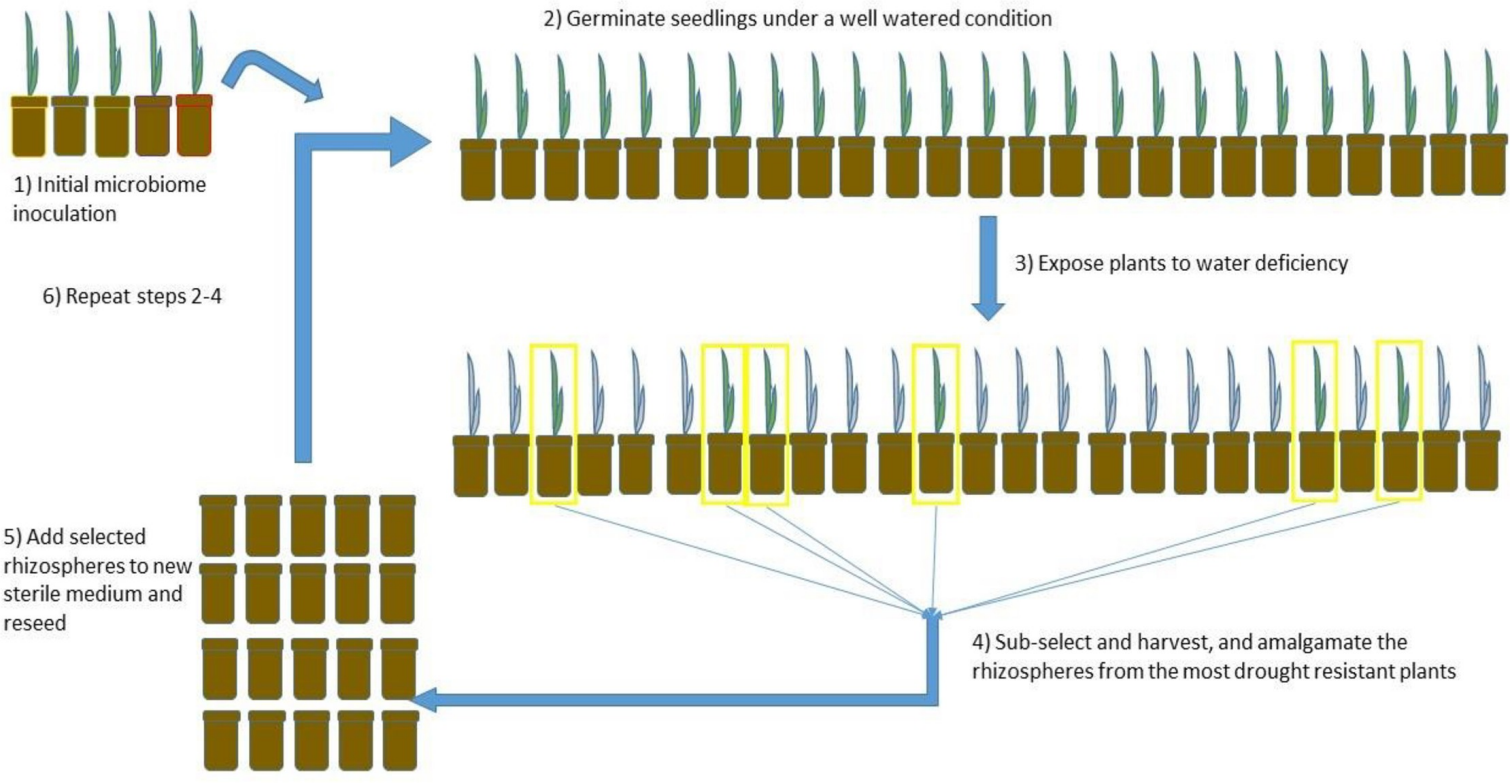
Concept of host mediated microbiome engineering (HMME). 1) An initial microbiome is inoculated. 2) Seeds are planted into well-watered conditions. 3) Emerging seedlings are then exposed to a drought stress by withholding watering. 4) When 90% of the plants display symptoms of water stress (wilting, leaf curling, etc.), the 5 best-performing plants are selected. Their rhizospheres (roots and planting medium) are amalgamated with autoclaved Metro-Mix 900 in a 1:10 ratio. The next round of selection is then initiated by planting seeds into the engineered planting medium.

Host-mediated microbiome engineering (HMME) is a cycle-dependent strategy that indirectly selects microbiomes based on host phenotype (Fig 1). For example, by directly selecting for increased seedling water stress tolerance, the host phenotype (e.g., delayed onset of seedling water deficit stress symptoms) is used to indirectly select for beneficial microbiome-host interactions over multiple generations using the same host germplasm. In this host-centric selection process, all microbiota are sub-selected at a community level, rather than on an individual basis [1]. This method allows microbiomes to change through both ecological (e.g., diversity, relative abundance) and evolutionary (e.g., extinction events, alterations in allele frequency, mutation, horizontal gene transfer) processes [2]. Previous research demonstrated that HMME can indirectly select microbiomes for enhanced growth under altered soil pH by utilizing above ground biomass as a selection marker in *A*. *thaliana* [1]. Similarly, HMME was used to cultivate microbiomes capable of altering flowering onset and leaf biomass [3, 4]. In *Brachypodium distachyon*, results suggested HMME indirectly selected a rhizosphere microbiome that conferred salt-tolerance measured through the host phenotype [5].

In the present study, we sought to use HMME to improve wheat (*Triticum aestivum*) seedling establishment under severe water stress associated with lack of rainfall, since seedling establishment is often the most vulnerable stage and may have large impacts on crop stand and yield [6]. The host phenotype used for screening was the delayed of onset of drought stress symptoms in wheat seedlings establishing under waster deficit conditions. The source of the original was obtained from the rhizospheres of perennial grasses collected from El Paso, TX, where the semi-arid environment provides a strong selective pressure for survival under nearly constant water deficit. The rationale for choosing the starting material was that perennial grasses growing vigorously under pervasive water stress conditions were likely to foster a microbiome capable of mediating drought stress. In this HMME experiment, seeds of wheat cultivar TAM 111 were planted into well-watered planting medium inoculated with the microbiome from the grassland rhizosphere soil. Water was then withheld until 90% of the seedlings showed symptoms of extreme drought stress (wilting to collapse). The plants displaying the least drought stress were selected and their rhizospheres (roots and planting medium) were then used as inoculum for subsequent selection cycles. Each of the subsequent selection cycles were similarly halted when 90% of the seedlings experienced collapse and again the rhizospheres of the best performing plants were used as inoculum. The cycling was terminated when there were no further improvements in time to wilting. Additional objectives of the study were to 1) determine whether changes in plant growth and development were associated with HMME-mediated improvements in water stress tolerance by comparing plants grown with non-autoclaved versus autoclaved inoculum from the final round of selection and 2) characterize changes in the taxonomic and functional diversity of the wheat seedling microbiomes during HMME selection rounds.

## Methods

### Rhizobiome sampling

Twenty-five bermudagrass (*Cynodon* spp.) thatch core samples (10 cm diameter, 15 cm depth) were collected alongside medians, parks, roadsides, and ranches in the summer of 2016 in El Paso, Texas, USA. There were no specific permissions required for the locations where bermudagrass samples were collected, since those places were public area. Bermudagrass is well established in these areas and is not endangered or protected species. Intact core samples were immediately shipped upon removal under ambient temperatures to College Station, TX. Each sample core was then subdivided into 5 cm diameter cores, transferred to a round plastic pot (10 cm diameter, 8 cm height), filled-in with autoclaved potting mix (Metro-Mix 900, Sun Gro Horticulture, Agawam, MA), and grown in a greenhouse for 14 days. Grasses were exposed to three different levels of watering: non-stressed (watering up to the field capacity every other day), moderate stress (watering once a week), and severe stress (no watering). The onset of drought symptoms was monitored and recorded based on plant phenotype: leaf wilting, curling, tip burning, and plant lodging. The five cores containing plants for which drought symptoms were most delayed under both the moderate and severe watering regimes were used for the following microbiome engineering experiment.

### Host-mediated microbiome engineering

The entire root system from the selected grasses were separated from aboveground tissue and the root system and soil from the container were used as the inoculum for the initial HMME (Round 0). The inoculum was combined with autoclaved potting medium (Metro-Mix 900) at a 1:10 ratio (soil: potting mix by volume), watered to saturation, and mixed thoroughly in a sterile autoclave bag. A total of 50 pots (10 cm diameter, 8 cm depth) each filled with 400 ml (by volume) of the amalgamated medium. Wheat (*Triticum aestivum* subsp. *aestivum* cultivar TAM111) seeds were surface sterilized in 10% NaOCl for 10 min, followed by 10 subsequent washes in sterile dH_2_O. Seeds were germinated on sterile filter paper 24 hr at 37°C. On day 0, each pot was sown with 5 seeds and covered lightly. Pots were incubated without any further watering in a growth chamber at 30°C, using fluorescent bulbs emitting 300 μmol m^-2^ s^-1^, 12 hr:12 hr light / dark cycle. When 90% (45 out of 50) of the pots had plants displayed symptoms of severe water stress (wilting to collapse), the 5 pots containing the best-performing plants were selected. The rhizospheres (roots and planting medium) from the selected pots were then amalgamated with fresh, autoclaved Metro-Mix 900 in a 1:10 ratio. The amalgamated medium was again watered to saturation and mixed thoroughly. The next round of selection was initiated by planting seeds into the engineered planting medium. The artificial selection cycles continued to the point when the number of days delay in the onset of drought symptoms was no longer increasing (Fig 1).

### Plant phenotype

To determine the effect of HMME on plant growth and development, germinated wheat seeds were planted into medium containing non-autoclaved or autoclaved inoculum (control) from the final round of selection (e.g. 1:10 ratio inoculum to autoclaved Metro-Mix 900). Pots were watered to field capacity, given no additional watering, and maintained under the aforementioned growth chamber conditions. After 10 days, plants were harvested, and roots were washed on a fine mesh sieve to remove debris. Whole plant fresh weight (biomass) was measured, and then roots were separated from above ground tissue and scanned using a flatbed scanner (EPSON, Perfection V-750). Intact root systems were transferred to a root positioning tray (20 cm × 30 cm) with sterile water (three root systems per tray) and carefully spread out to avoid root overlap. Root scans were analyzed using WinRHIZO Arabidopsis 2017a (Regent Instruments Inc., Quebec, Canada, 2000), generating estimates of total root length, root surface area, and number of root tips as previously described [7, 8]. Dry weights were obtained after scanning and compared as previously described [9], This experiment was repeated once.

### Effect of dilution of HMME inoculum

Rhizosphere inoculum from the final round of HMME was serially diluted to 10^−1^, 10^−2^ and 10^−3^ by amalgamation with non-autoclaved potting mix, Metro-mix 900. Metro-mix 900 receiving no HMME inoculum was used as the control. Treatments were wetted, mixed, and added to pots, and seeds were planted as described previously. The pots received no further water and were incubated under the aforementioned growth chamber conditions. Plant water stress symptoms were compared among treatments on day 10. To determine water loss over time, pots were weighed every 48 hrs. and the percentage water loss was calculated as the change over 48 hr. periods standardized to initial pot weight.

### Statistical analyses

Treatments were arranged in a completely randomized block design. Differences among HMME inoculation treatments in plant growth and development traits, percent water loss, and OTU table relative abundances were analyzed by analysis of variance (ANOVA) using SAS version 9.3 software (SAS Inc., Cary, NC). Pairwise comparisons between the treatments were analyzed using a protected Fisher’s least significant difference (LSD) test.

### DNA extraction and 16s rRNA sequencing

Genomic DNA from rhizosphere samples were extracted using the ZymoBIOMICS^®^ DNA Miniprep Kit, SKU D4300 (Zymo Research). A control sample using the ZymoBIOMICS^®^ Microbial Community Standard was also used as a positive control for detecting bias and background contamination. DNA samples were then sent to Novogene (Novogene Corporation, Inc.) for PCR amplification of the V4 region of 16S rRNA using primer set of 515f (5’-GTGCCAGCMGCCGCGGTAA-3’) and 806r (5’-GGACTACHVGGGTWTCTAAT-3’) followed by next generation sequencing (NGS) on an illumina HiSeq 4000 [10]. Sample prep, PCR, library prep, and NGS sequencing were done in accordance with Novogene protocols (Novogene Inc.) [11–13]. Generated paired-end 250 bp raw reads and metadata were uploaded to NCBI (SRA: SRP158143, BioProject: PRJNA486342).

### Bioinformatic processing and analysis

All of the analyses were conducted in the command line environment and executed on the Ada supercluster at the high-performance research computing center (HPRC) at Texas A&M University. Barcodes were removed from the NGS raw reads via the bioinformatics software package Trimmomatic [14] and imported into QIIME 2 Core 2018.6 (www.qiime2.org) using q2cli interface. Inside the QIIME2 environment, forward and reverse sequences were merged, and paired-end sequences were denoised, dereplicated, and filtered for chimeras using the Divisive Amplicon Denoising Algorithm (DADA2) plugin [15]. Following DADA2, the sequences were aligned using MAFFT, masked for highly variable sequences using qiime2 mask, and converted into a phylogenetic tree using the QIIME2 diversity plugin pipeline for exploring community diversity, with a subsampling depth of 1109 determined by preliminary analysis of the feature table. The diversity pipeline analyzed sequences for core-metrics results, ANOVA, alpha diversity, beta diversity, and constructed principle component of analysis (PCoA) plots using Bray-Curtis dissimilarity and weighted UniFrac distances. After diversity analysis, the sequences were feature classified into organizational taxonomic units (OTUs) using the 99% Greengenes 13_8 reference database (greengenes.microbio.me/greengenes_release/) for the 515f/806r, visualized using taxa bar plots, and collapsed into a table for each taxa level.

## Results

**Fig 2:**
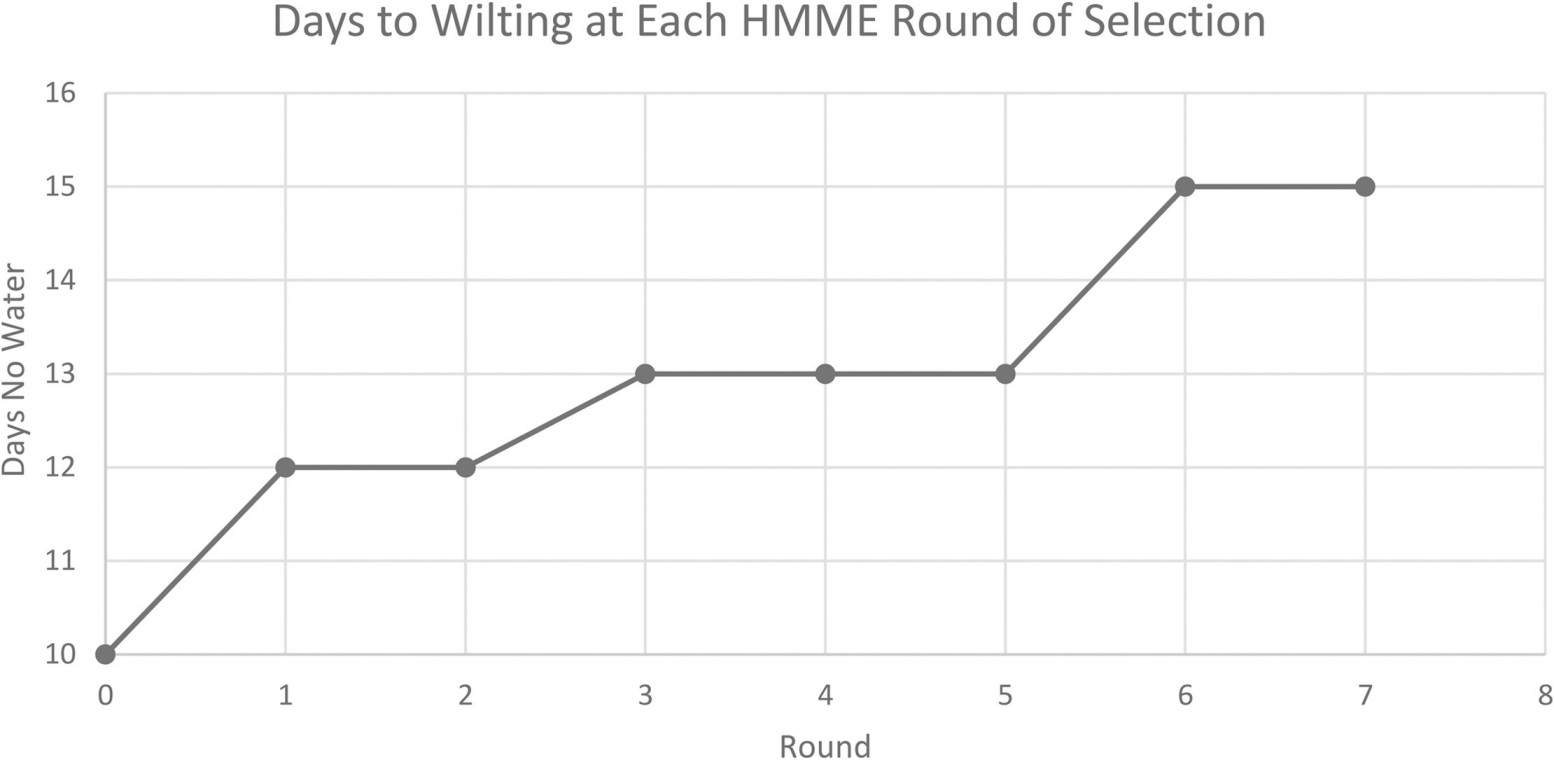
Effect of host-mediated microbiome engineering (HMME) rounds of selection on seedling drought tolerance. The number of days without water was determined as the day on which 90% of the seedlings displayed severe water stress symptoms.

**Fig 3:**
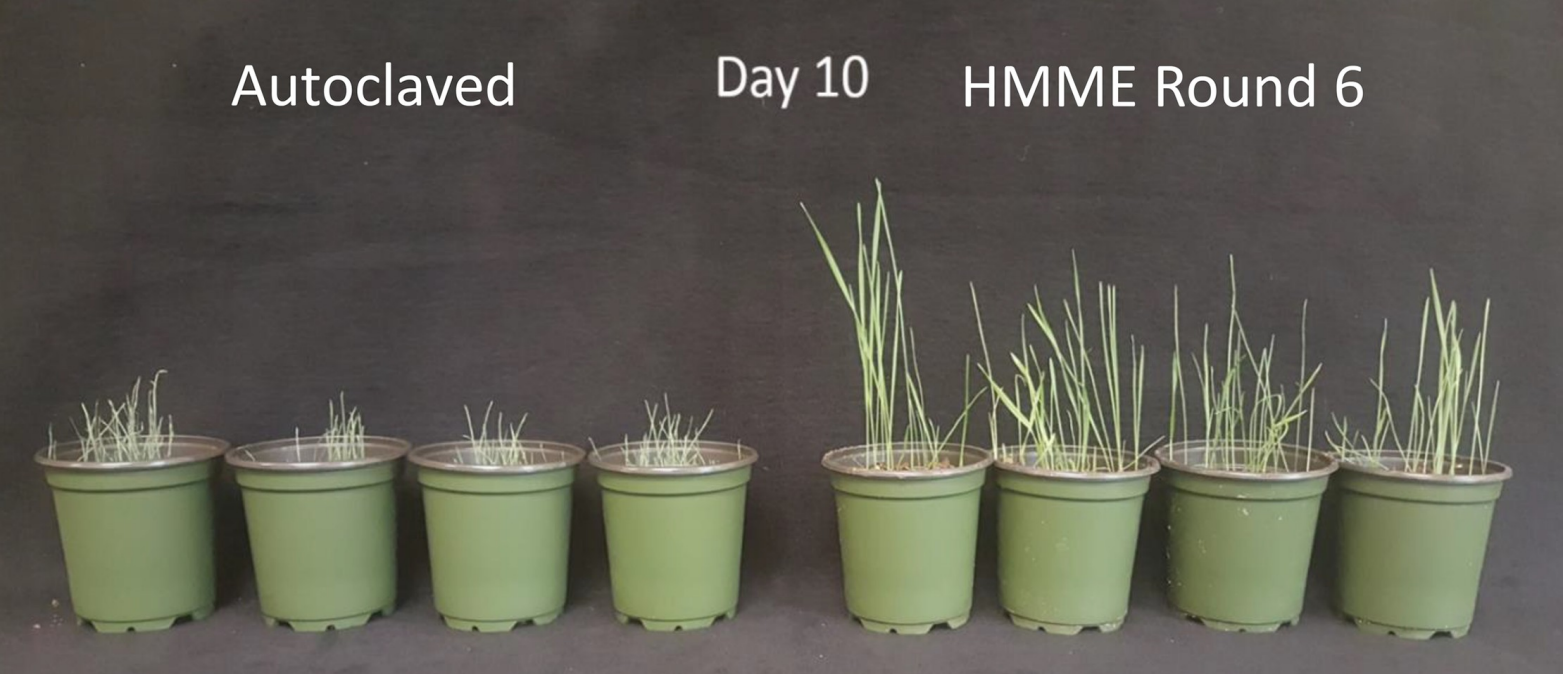
Effect of host-mediated microbiome engineering (HMME) on seedling phenotypes under drought stress. Wheat seedlings growing in planting medium combined with either autoclaved (left) or non-autoclaved (right) rhizosphere inoculum from the final HMME round of selection after 10 days of withholding water.

**Fig 4:**
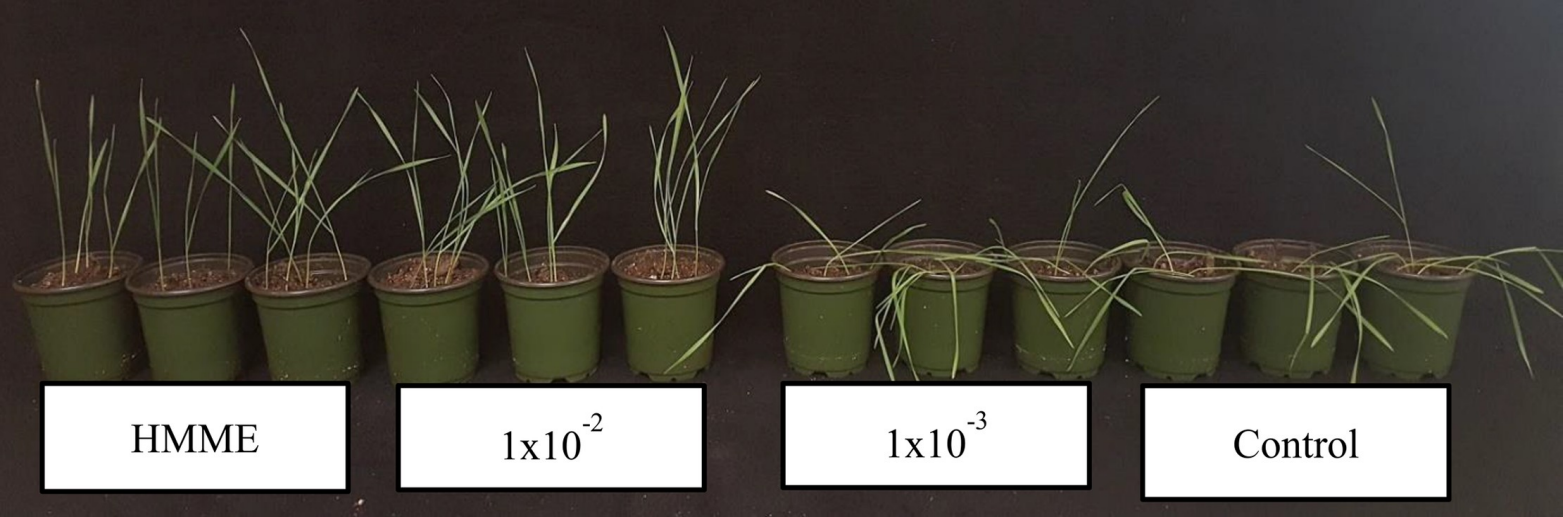
Transferability of the host-mediated microbiome engineering (HMME) effect on plant water stress tolerance. Dilution of rhizosphere inoculum from HMME Round 6 demonstrates no loss of effectiveness in mediating the onset drought stress symptoms at day 10 for the 1x10^-1^ (HMME) and 1x10^-2^ dilution, but loss of efficacy at the 1x10^-3^ dilution, which displayed a similar phenotype to the treatment having no HMME inoculum (control).

**Fig 5:**
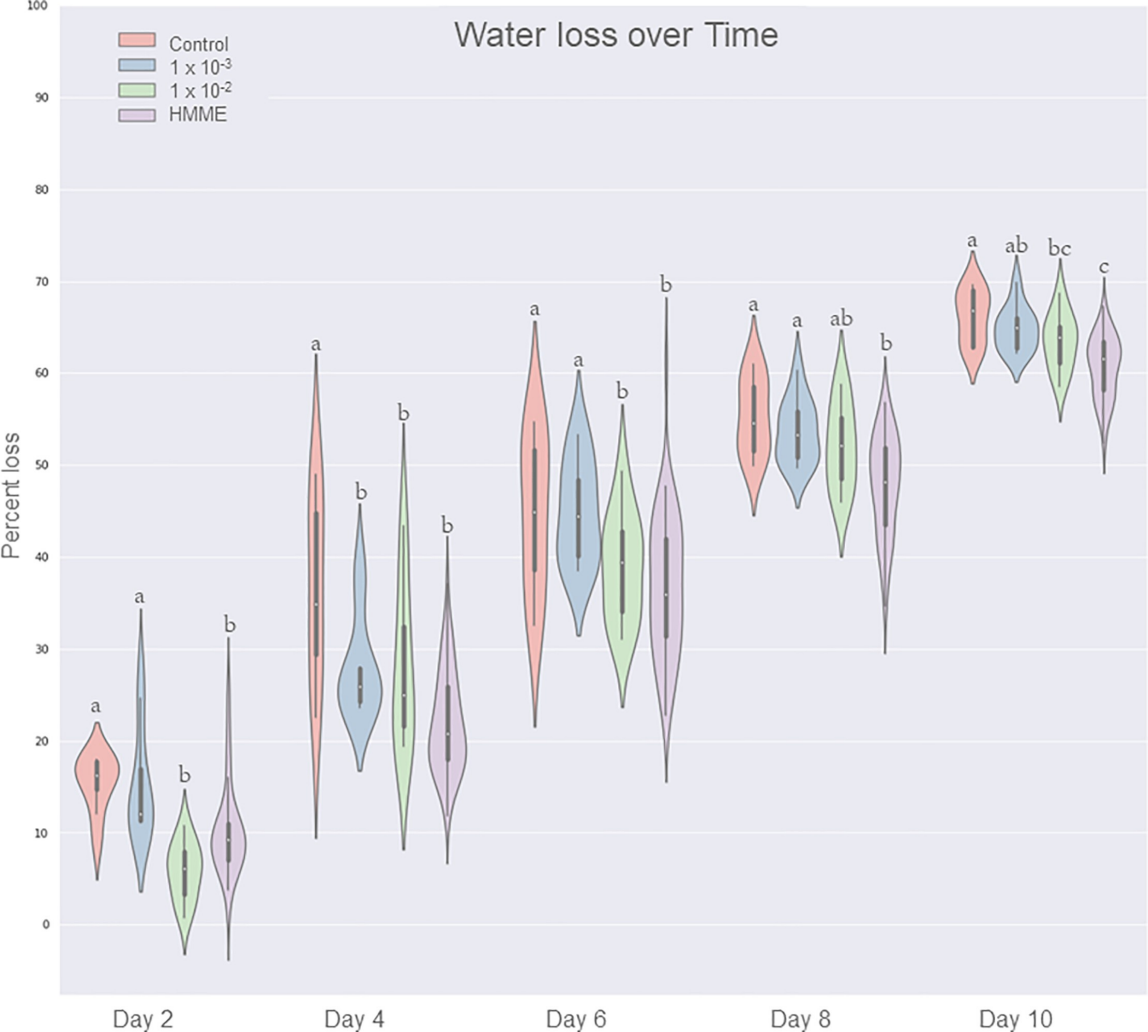
Transferability of the host-mediated microbiome engineering (HMME) effect on water loss. Percentage of water loss calculated from the difference in pot weight every 48 hrs. relative to the starting pot weight. Treatments include pots growing seedlings inoculated with either a dilution series of rhizosphere inoculum from HMME Round 6: 1x10^-1^ (HMME), 1x10^-2^, 1x10^-3^, or no HMME inoculum (control). Standard error bars are shown, and significant differences are indicated.

After six rounds of artificial selection using HMME, wheat seedlings exhibited a 5-day delay in the onset of drought stress symptoms compared to Round 0 (Fig 2). There were no differences in the number of days to the onset of drought symptom between Rounds 6 and 7. This water stress tolerance phenotype was abolished when plants were grown in medium amended with steam autoclaved HMME inoculum (Fig 3) or potting mix containing no HMME inoculum (Fig 4), indicating that this phenotype was derived from a microbiome effect. Plants grown with HMME inoculum had significantly greater plant biomass, root dry weight, and total root length and surface area when compared to plants grown with autoclaved HMME inoculum (Table 1, Table 2). The effect of HMME inoculum on plant water stress tolerance was transferable at 10^−1^ and 10^−2^ dilutions but lost at the 10^−3^ dilution (Fig 4). On day 0, pot weights were significantly (*p* ≥ 0.05) greater for pots with plants grown in the 10^−1^ dilution of inoculum as compared to pots with plants grown in the 10^−3^ dilution or control inoculum (10^−1^ = 293.59 g ± 2.11; 10^−2^ = 279.28 g ± 4.09; 10^−3^ = 279.28 g ± 5.12; and control = 280.10 g ± 2.11). These differences in initial pot weight confirm visual observations made following medium saturation and mixing that the HMME inoculum appeared to have enhanced soil aggregation and water holding capacity. Over the ten-day drying period, the percentage water loss from pots was generally less for treatments with more HMME inoculum. The difference in percent water loss was most apparent at days 4 and 6, where the average water loss for the HMME treatment was 22.39% and 37.23% compared to 36.90% and 44.91% for the control, respectively (Fig 5, S1 Table).

**Table 1:** Analysis of variance (ANOVA) for testing the effect of the host-mediated microbiome engineering (HMME) on plant phenotype under drought stress.

Dependent Variable	df	Mean squared	*F*	*P*
Root length (RL)	1	837.022407	10.15	0.0097
Total Biomass (FW)	1	17572.05333	39.04	< 0.0001
Root Dry Weight (RDW)	1	9.90083333	6.82	0.0260
Root Surface Area (RSA)	1	6.70657008	11.04	0.0077
Number of root tips (TIPS)	1	5940.75000	2.79	0.1258

**Table 2:** Comparison of root system traits for plants grown with either non-autoclaved (HMME) or autoclaved HMME inoculum (control).

Treatment	Root Length (cm)	Biomass (mg)	Root Dry Weight (mg)	Root Surface Area (cm^2^)	Tips
HMME	64.67 B	235.13 B	6.43 B	6.52 B	215.67
Control	47.97 A	158.60 A	4.62 A	5.02 A	171.17

The different letters within the column indicates significant difference between treatments at Fisher’s LSD test at *P* = 0.05.

### Taxonomic analysis

**Fig 6:**
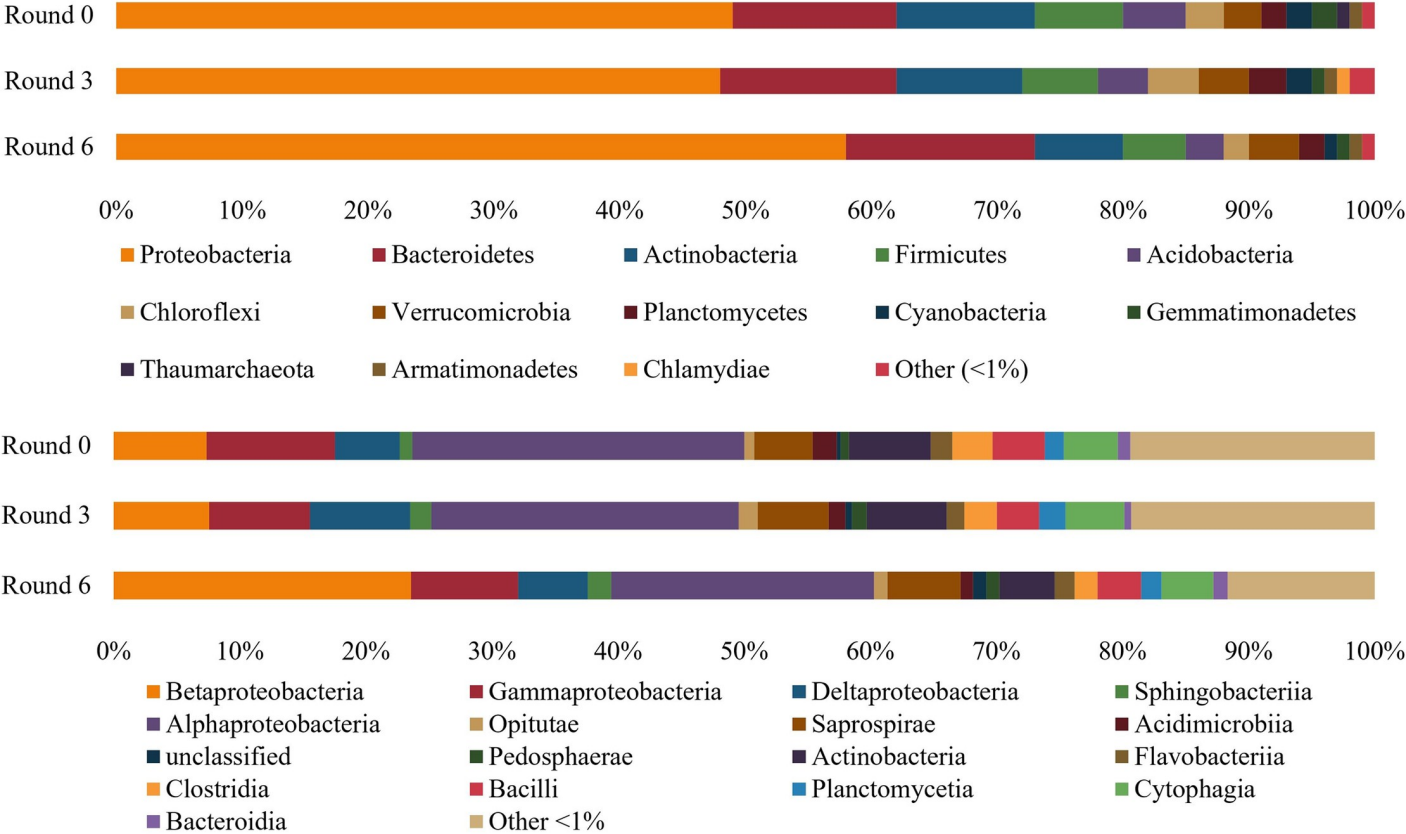
Taxonomic analysis. Stacked bar charts presenting relative abundance of each taxa at the phylum level (top) and class level (bottom). This comparison demonstrates host-mediated microbiome engineering (HMME) resulted in taxonomic increases in Proteobacteria and Betaproteobacteria in the community rhizosphere when comparing Round 0, 3 and 6 of HMME. Taxa were referenced to the 99% OTU Greengenes 13–8 database with a 1% abundance cutoff.

Results from the 16S rRNA amplicon sequencing revealed changes in relative microbial taxon abundance at both the phylum and class levels (Fig 6, S1 Fig). The greatest change was the increase from 49.2% to 59.1% in the relative abundance of phylum proteobacteria when comparing Round 0 (R0) to Round 6 (R6) (Table 3). In contrast, when comparing R0 to R6, the relative abundance of actinobacteria (10.9% R0 to 6.2% R6) and acidobacteria (4.8% R0 to 2.4% R6) both decreased (Table 3). At the class level (Fig 6), there was over a threefold increase in the relative abundance of betaproteobacteria (7.3% R0 to 23.6% R6), a decrease in gammaproteobacteria (10.2% R0 to 8.5% R6), and marginal decreases in alphaproteobacteria (26.3% R0 to 20.8% R6) (Table 4).

**Table 3:** Phylum level analysis of variance (ANOVA) comparing Rounds 0, 3, and 6 of host-mediated microbiome engineering (HMME).

Phylum	Round 0	Round 3	Round 6	*P*-value
Proteobacteria	49.2	48.4	59.1	0.0670
Bacteroidetes	13.6	15.0	14.9	0.7130
Actinobacteria	10.9	9.0	6.2	0.1051
Firmicutes	7.4	6.0	5.4	0.4537
Verrucomicrobia	2.7	4.1	3.3	0.2004
Acidobacteria	4.8	4.2	2.4	0.0371
Chloroflexi	3.3	3.9	2.1	0.0743
Planctomycetes	1.8	2.8	2.0	0.3821
Cyanobacteria	1.7	1.7	1.5	0.9697
Gemmatimonadetes	1.5	1.3	0.7	0.0304

**Table 4:** Class level analysis of variance (ANOVA) comparing Rounds 0, 3, and 6 of host-mediated microbiome engineering (HMME).

Class	Round 0	Round 3	Round 6	*P*-value
Betaproteobacteria	7.3	7.6	23.6	0.008
Gammaproteobacteria	10.2	8.0	8.5	0.031
Deltaproteobacteria	5.1	8.0	5.5	0.055
Alphaproteobacteria	26.3	24.4	20.8	0.103

### Phylogenetic diversity

**Fig 7:**
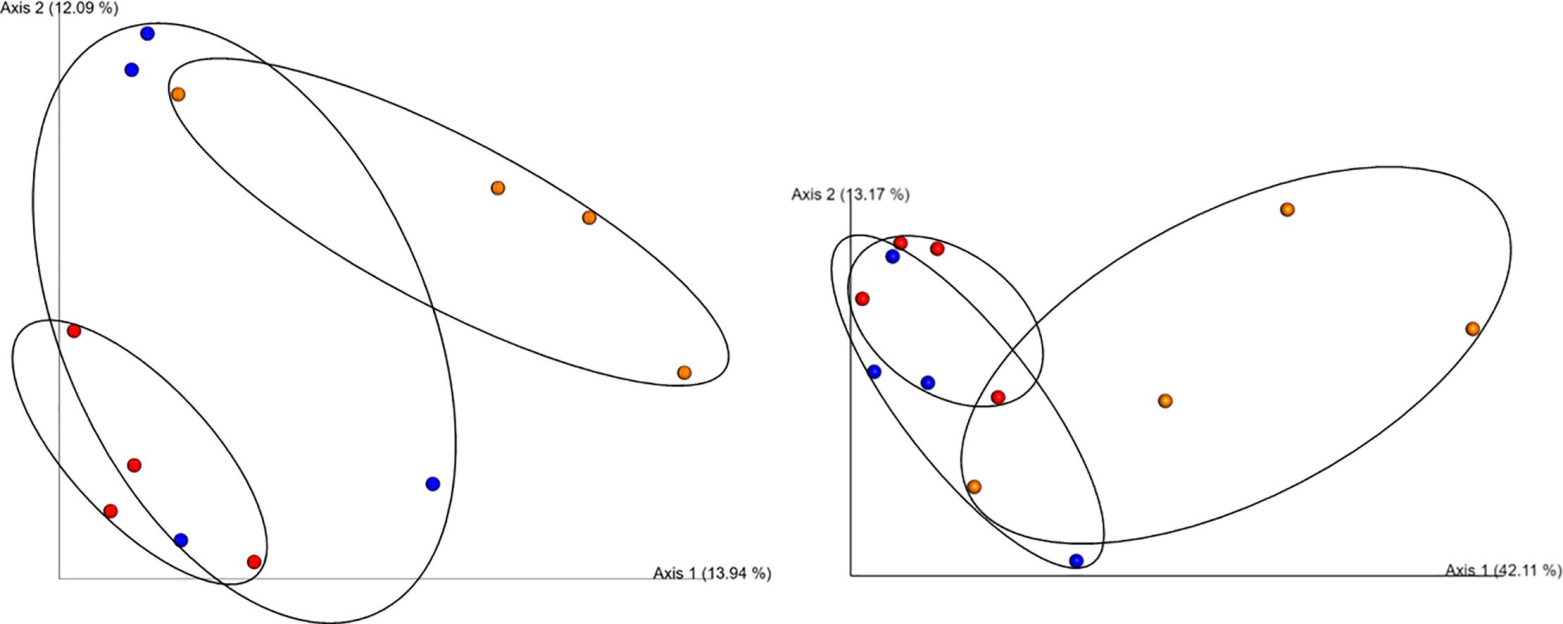
Beta-diversity. When comparing both a Bray Curtis dissimilarity index (A) and a Weighted UNIFRAC PCoA plot (B), successive generations show increasing divergence or dissimilarity from the original community extracted from Round 0 (red), with Round 3 (blue) transitioning between Round 0 and Round 6 (orange) of host-mediated microbiome engineering (HMME).

Alpha diversity Simpson correlation using a Spearman’s test statistic resulted in a significant decrease in alpha diversity when comparing R0 to R6 (*p* = 0.048). Beta diversity analysis using a PERMANOVA pseudo-*F* associated analysis with 999 permutations revealed a statistically significant difference when comparing R0 and R6 (*p* = 0.029), but not between R0 and R3 or R3 and R6 (Table 5). Beta diversity PCoA plots using a Bray Curtis dissimilarity index or a Weighted UNIFRAC index revealed dissimilarity in overall bacterial OTU composition between R0 and R6 with R3 transitioning between the two (Fig 7).

**Table 5:** Comparative analysis of Rounds 0, 3, and 6 of host-mediated microbiome engineering (HMME) using a PERMANOVA pseudo-*F* association analysis (permutations = 999).

Group 1	Group 2	Sample size	Permutations	pseudo-*F*	*P*-value
Round 0	Round 3	8	999	1.165669	0.177
Round 0	Round 6	8	999	1.407776	0.029
Round 3	Round 6	8	999	1.200876	0.126

## Discussion

To our knowledge, this study provides the first example of utilizing HMME to indirectly select a rhizosphere microbiome that increased the seedling drought tolerance during water deficit conditions, by directly selecting for a delay in the onset of drought symptoms in newly established wheat seedlings. A steady increase in the ability of plants to survive water deficit occurred from the start of the experiment over the 6 rounds of HMME selection. Drought stress symptom onset delayed from 10 to 15 days. The improvements in drought tolerance stabilized after 6 rounds of HMME selection (and thus 6 rounds of 1:10 dilutions), and was transferable in subsequent 1:100 dilution, but lost if diluted further. The effect on seedling water stress tolerance was abolished by autoclaving the HMME inoculum. These results indicate that the change in plant adaptation to drought stress was a function of microbial population dynamics.

The single greatest challenge in this HMME experiment lied in the experimental design, as it is impossible to separate soil components with microbes completely. Ideally, the negative control for the HMME study would have same rhizosphere components except microbes but reproducing this negative control treatment is extremely difficult to produce due to the iterative temporal nature of the study. We instead used the autoclaved HMME soil as the negative control to compare with the HMME soil and determine the effect of selected microbial community in the HMME soil on the host plants. However, we could not exclude any unknown effects from autoclaving soil. Alternatively, we also conducted the experiment that the HMME soil was diluted with non-autoclave potting mix to verify the effect of selected microbiomes. Previous studies have also utilized a “low-line” treatment consisting of the worst-performing pots as a control for the HMME experiment [1, 2, 4]. During the experimental design stage of our experiment, we identified that incorporating a low-line HMME soil selected based on a low-scoring plant phenotype in our screening method also caused the accumulation of pathogens in the soil, as previous reports have suggested [1, 4, 16]. Picking the worst-performing pots to carry through the continuous rounds of the HMME process resulted in severe diseases or complete wilts of tested plants due to the generational recruitment of plant pathogens. We agree that the low-line microbiome would have been a logically idealistic control, but the low-line soil that easily recruits and becomes dominated by pathogenic microbes could not serve as a control in our HMME study. Given these limitations at preparing the control soils to compare the HMME soil with, we were able to determine the effect of the HMME soil by two different methods, the comparison with autoclaved HMME soil and the serial dilution assay of the HMME soil using non-autoclaved potting mix.

The increase in seedling drought tolerance with HMME was accompanied by changes in seedling growth and development. Seedlings grown with HMME inoculum were larger in size and had more extensive root system development than seedlings grown with autoclaved HMME inoculum. The observed alterations in plant growth and root system development are consistent with plant adaptation for maintaining plant productivity under drought [17]. Previous research showed that increased root length and surface area contribute to increased soil exploration for available water [18–21]. It is unclear the extent to which the HMME microbiome may have contributed directly to plant growth and development via plant growth promoting activities and/or indirectly via modifications to the rhizosphere environment. For example, plant growth-promoting bacteria have been reported to contribute to plant growth and root development via a number of mechanisms including suppression of seedling disease (although not a factor in this experiment) [22], production of plant growth regulating compounds [23–25], assistance in nutrient uptake [26], and mediation of redox stress [18]. Additionally, previous research has demonstrated that extracellular polysaccharide production from beneficial microbiota provides significant indirect benefits to plant growth and development via improved soil structure and increased soil water retention [21, 27]. These improvements coupled with higher respiration rates (and associated water release) by microbial communities selected for rapid growth and colonization of wheat seedling rhizospheres may lead to further improvements in water availability in the engineered microbiomes, therein enabling plants to avoid drought stress longer. Indeed, we observed (both visually and in terms of weight/volume) greater water retention in the HMME soil at the start and throughout the experiment.

Each round of HMME was associated with changes in taxonomic diversity and composition. As expected, dilution with HMME rounds of selection resulted in a reduction in alpha diversity [28]. Comparison of beta diversity indicated potential host-mediated changes in rhizosphere populations with successive generations, showing increasing divergence or dissimilarity from the original community composition. Similar trends in species community structure associated with dilution were noted previously, although functional profiles of rhizosphere communities resulting from dilution overlapped more [28]. Explaining this trend, the authors hypothesized that enrichment processes in the rhizosphere were more likely to select microbes with particular functionalities than taxonomies. The observed changes in relative abundance in Actinobacteria and Betaproteobacteria with each round of selection (S1 Fig) is a unique observation not typically seen in other drought related microbiome research studies [23, 25, 29–31]. Reasoning for the differences in this observation are currently unknown but may lie in the comparison of the differences in the objective and experimental design of the study. Our study of the wheat seedling rhizosphere microbiome under drought conditions consisted of an iterative microbiome propagation and differentiation strategy focused on ascertaining an increased understanding of an optimized microbiome to withstand seedling drought through simultaneous manipulation of ecological and evolutionary pressures. Previous research on root microbiomes associated with water stress utilize different environmental conditions, different mechanisms for implementing water stress, different stages of growth and different plant models. The most notable difference of our experiment from previous research is the integration of a microbiome selection, transplantation and propagation-based strategy, focusing on the phenotype of the host as a selection marker.

In another study, we conducted an analysis of predicted functional metagenomic changes using the bioinformatic software package Phylogenetic Investigation of Communities by Reconstruction of Unobserved States (PICRUSt) [32]. Results from this analysis yielded statistically significant increases level 2 KEGG orthologs involved in metabolism, signal transduction, cell processes and signaling, and cell motility when comparing across the R0 to R6 [33]. Pairwise comparisons between R0 and R6 with 95% confidence revealed similar significant increases in the KEGG orthologs associated with metabolism, signal transduction, cell processes and signaling, and cell motility [33]. The average nearest sequenced taxon index (NTSI), which reflect the relatedness to the reference genomes, were all less than 0.15 (> 85% similarity), indicating acceptable inference data quality [32]. Although this inference is at too coarse of a scale to draw many conclusions, enrichment in motility and metabolism are consistent with selection for a microbiome that can colonize and proliferate quickly (e.g., within 10 to 15 days of germination) in the seedling rhizosphere as would be selected by our HMME protocol.

## Conclusions

In summary, we showed that HMME can be used to enhance seedling drought tolerance at least under our experimental conditions. The findings from this study support the hypothesis that host mediated microbiome engineering can be used to alter the root rhizosphere microbiome and demonstrate the potential of engineered microbiomes to mediate changes in the rhizosphere environment, effectuating improved plant adaptation to water stress. Future research to test these observations should include mechanistic studies that profile the plants under duress from water deficit, exopolysaccharide production characterization assays of the microbiomes from each generation, and shotgun metagenomic sequencing for hologenome characterization and the observation of plasmid exchange, horizontal gene transfer, and changes in allelic frequency.

## Supporting information

S1 Table Effect of dilution of host-mediated microbiome engineering (HMME) inoculum on water loss. Over the ten-day drying period, water loss was measured from the different soils including rhizosphere inoculum from the 6 round of HMME diluted to 10^−1^, 10^−2^, 10^−3^ by non-autoclaved potting mix and the control potting mix receiving no HMME inoculum.
(XLSX)

S1 Fig Relative abundance of major bacteria in Actinobacteria, Bacteroidetes, and Proteobacteria at Round 0, 3, and 6 of host-mediated microbiome engineering (HMME). (JPG)

## References

[1] Swenson W, Wilson DS, Elias R. Artificial ecosystem selection. Proc Natl Acad Sci U S A. 2000;97(16):9110–4. Epub 2000/07/13. 10.1073/pnas.150237597 10890915 PMC16830

[2] Mueller UG, Sachs JL. Engineering microbiomes to improve plant and animal health. Trends Microbiol. 2015;23(10):606–17. Epub 2015/10/01. 10.1016/j.tim.2015.07.009 . 26422463

[3] Panke-Buisse K, Lee S, Kao-Kniffin J. Cultivated sub-populations of soil microbiomes retain early flowering plant trait. Microb Ecol. 2017;73(2):394–403. Epub 2016/09/23. 10.1007/s00248-016-0846-1 27655524 PMC5272889

[4] Panke-Buisse K, Poole AC, Goodrich JK, Ley RE, Kao-Kniffin J. Selection on soil microbiomes reveals reproducible impacts on plant function. ISME J. 2014;9:980 10.1038/ismej.2014.196 https://www.nature.com/articles/ismej2014196#supplementary-information. 25350154 PMC4817706

[5] Mueller UG, Juenger T, Kardish M, Carlson A, Burns K, Smith C, et al Artificial microbiome-selection to engineer microbiomes that confer salt-tolerance to plants. bioRxiv. 2016:081521 10.1101/081521 PMC8631316 34846165

[6] Pessarakli M. Handbook of plant and crop stress 2nd ed. New York: M. Dekker; 1999. xviii, 1254 p. p.

[7] Himmelbauer ML, Loiskandl W, Kastanek F. Estimating length, average diameter and surface area of roots using two different Image analyses systems. Plant Soil. 2004;260(1–2):111–20. 10.1023/B:Plso.0000030171.28821.55 WOS:000221763000011.

[8] Arsenault J-L, Poulcur S, Messier C, Guay R. WinRHlZO^™^, a root-measuring system with a unique overlap correction method. HortScience. 1995;30(4):906.

[9] Garnier E. Growth analysis of congeneric annual and perennial grass species. J Ecol. 1992;80(4):665–75. 10.2307/2260858 WOS:A1992KF83700007.

[10] Thompson LR, Sanders JG, McDonald D, Amir A, Ladau J, Locey KJ, et al A communal catalogue reveals Earth’s multiscale microbial diversity. Nature. 2017;551:457 10.1038/nature24621 https://www.nature.com/articles/nature24621#supplementary-information. 29088705 PMC6192678

[11] Cock PJ, Fields CJ, Goto N, Heuer ML, Rice PM. The Sanger FASTQ file format for sequences with quality scores, and the Solexa/Illumina FASTQ variants. Nucleic Acids Res. 2010;38(6):1767–71. Epub 2009/12/18. 10.1093/nar/gkp1137 20015970 PMC2847217

[12] Hansen KD, Brenner SE, Dudoit S. Biases in Illumina transcriptome sequencing caused by random hexamer priming. Nucleic Acids Res. 2010;38(12):e131 Epub 2010/04/17. 10.1093/nar/gkq224 20395217 PMC2896536

[13] Erlich Y, Mitra PP, delaBastide M, McCombie WR, Hannon GJ. Alta-Cyclic: a self-optimizing base caller for next-generation sequencing. Nat Methods. 2008;5(8):679–82. Epub 2008/07/08. 10.1038/nmeth.1230 18604217 PMC2978646

[14] Bolger AM, Lohse M, Usadel B. Trimmomatic: a flexible trimmer for Illumina sequence data. Bioinformatics. 2014;30(15):2114–20. Epub 2014/04/04. 10.1093/bioinformatics/btu170 24695404 PMC4103590

[15] Callahan BJ, McMurdie PJ, Rosen MJ, Han AW, Johnson AJ, Holmes SP. DADA2: High-resolution sample inference from Illumina amplicon data. Nat Methods. 2016;13(7):581–3. Epub 2016/05/24. 10.1038/nmeth.3869 27214047 PMC4927377

[16] Santhanam R, Luu VT, Weinhold A, Goldberg J, Oh Y, Baldwin IT. Native root-associated bacteria rescue a plant from a sudden-wilt disease that emerged during continuous cropping. Proc Natl Acad Sci U S A. 2015;112(36):E5013–E20. 10.1073/pnas.1505765112 26305938 PMC4568709

[17] Comas LH, Becker SR, Cruz VM, Byrne PF, Dierig DA. Root traits contributing to plant productivity under drought. Front Plant Sci. 2013;4(442):442 Epub 2013/11/10. 10.3389/fpls.2013.00442 24204374 PMC3817922

[18] Ngumbi E, Kloepper J. Bacterial-mediated drought tolerance: Current and future prospects. Appl Soil Ecol. 2016;105:109–25. 10.1016/j.apsoil.2016.04.009 WOS:000377358300014.

[19] Barnawal D, Bharti N, Pandey SS, Pandey A, Chanotiya CS, Kalra A. Plant growth-promoting rhizobacteria enhance wheat salt and drought stress tolerance by altering endogenous phytohormone levels and TaCTR1/TaDREB2 expression. Physiol Plant. 2017;161(4):502–14. Epub 2017/08/09. 10.1111/ppl.12614 . 28786221

[20] Vardharajula S, Ali SZ, Grover M, Reddy G, Bandi V. Drought-tolerant plant growth promoting *Bacillus* spp.: effect on growth, osmolytes, and antioxidant status of maize under drought stress. J Plant Interact. 2011;6(1):1–14. 10.1080/17429145.2010.535178 WOS:000286491900001.

[21] Naseem H, Bano A. Role of plant growth-promoting rhizobacteria and their exopolysaccharide in drought tolerance of maize. J Plant Interact. 2014;9(1):689–701. 10.1080/17429145.2014.902125 WOS:000345150700050.

[22] Mendes R, Kruijt M, de Bruijn I, Dekkers E, van der Voort M, Schneider JH, et al Deciphering the rhizosphere microbiome for disease-suppressive bacteria. Science. 2011;332(6033):1097–100. Epub 2011/05/10. 10.1126/science.1203980 . 21551032

[23] Dimkpa C, Weinand T, Asch F. Plant-rhizobacteria interactions alleviate abiotic stress conditions. Plant Cell Environ. 2009;32(12):1682–94. Epub 2009/08/13. 10.1111/j.1365-3040.2009.02028.x . 19671096

[24] Dodd IC, Zinovkina NY, Safronova VI, Belimov AA. Rhizobacterial mediation of plant hormone status. Ann Appl Biol. 2010;157(3):361–79. 10.1111/j.1744-7348.2010.00439.x WOS:000283157700004.

[25] Timmusk S, Abd El-Daim IA, Copolovici L, Tanilas T, Kannaste A, Behers L, et al Drought-tolerance of wheat improved by rhizosphere bacteria from harsh environments: enhanced biomass production and reduced emissions of stress volatiles. PLoS One. 2014;9(5):e96086 Epub 2014/05/09. 10.1371/journal.pone.0096086 24811199 PMC4014485

[26] Yang J, Kloepper JW, Ryu CM. Rhizosphere bacteria help plants tolerate abiotic stress. Trends Plant Sci. 2009;14(1):1–4. Epub 2008/12/06. 10.1016/j.tplants.2008.10.004 . 19056309

[27] Chang WS, van de Mortel M, Nielsen L, Nino de Guzman G, Li X, Halverson LJ. Alginate production by Pseudomonas putida creates a hydrated microenvironment and contributes to biofilm architecture and stress tolerance under water-limiting conditions. J Bacteriol. 2007;189(22):8290–9. Epub 2007/07/03. 10.1128/JB.00727-07 17601783 PMC2168710

[28] Yan Y, Kuramae EE, de Hollander M, Klinkhamer PGL, van Veen JA. Functional traits dominate the diversity-related selection of bacterial communities in the rhizosphere. ISME J. 2017;11(1):56–66. 10.1038/ismej.2016.108 27482928 PMC5315473

[29] Ngumbi E, Kloepper J. Bacterial-mediated drought tolerance: Current and future prospects. Appl Soil Ecol. 2016;105:109–25. 10.1016/j.apsoil.2016.04.009 WOS:000377358300014.

[30] Fitzpatrick CR, Copeland J, Wang PW, Guttman DS, Kotanen PM, Johnson MTJ. Assembly and ecological function of the root microbiome across angiosperm plant species. Proc Natl Acad Sci U S A. 2018;115(6):E1157–E65. 10.1073/pnas.1717617115 29358405 PMC5819437

[31] Xu L, Naylor D, Dong Z, Simmons T, Pierroz G, Hixson KK, et al Drought delays development of the sorghum root microbiome and enriches for monoderm bacteria. Proc Natl Acad Sci U S A. 2018;115(18):E4284–E93. 10.1073/pnas.1717308115 29666229 PMC5939072

[32] Langille MG, Zaneveld J, Caporaso JG, McDonald D, Knights D, Reyes JA, et al Predictive functional profiling of microbial communities using 16S rRNA marker gene sequences. Nat Biotechnol. 2013;31(9):814–21. Epub 2013/08/27. 10.1038/nbt.2676 23975157 PMC3819121

[33] Jochum MD. Enhanced Drought Tolerance through Plant Growth Promoting Rhizobacteria and Microbiome Engineering Applications Texas A & M University 2019:107.

