# Supplementary figures and images for: Host-mediated microbiome engineering (HMME) of drought tolerance in the wheat rhizosphere
Source: PLoS One. 2019 Dec 4;14(12):e0225933. doi: 10.1371/journal.pone.0225933 (PMC6892483; doi:10.1371/journal.pone.0225933)

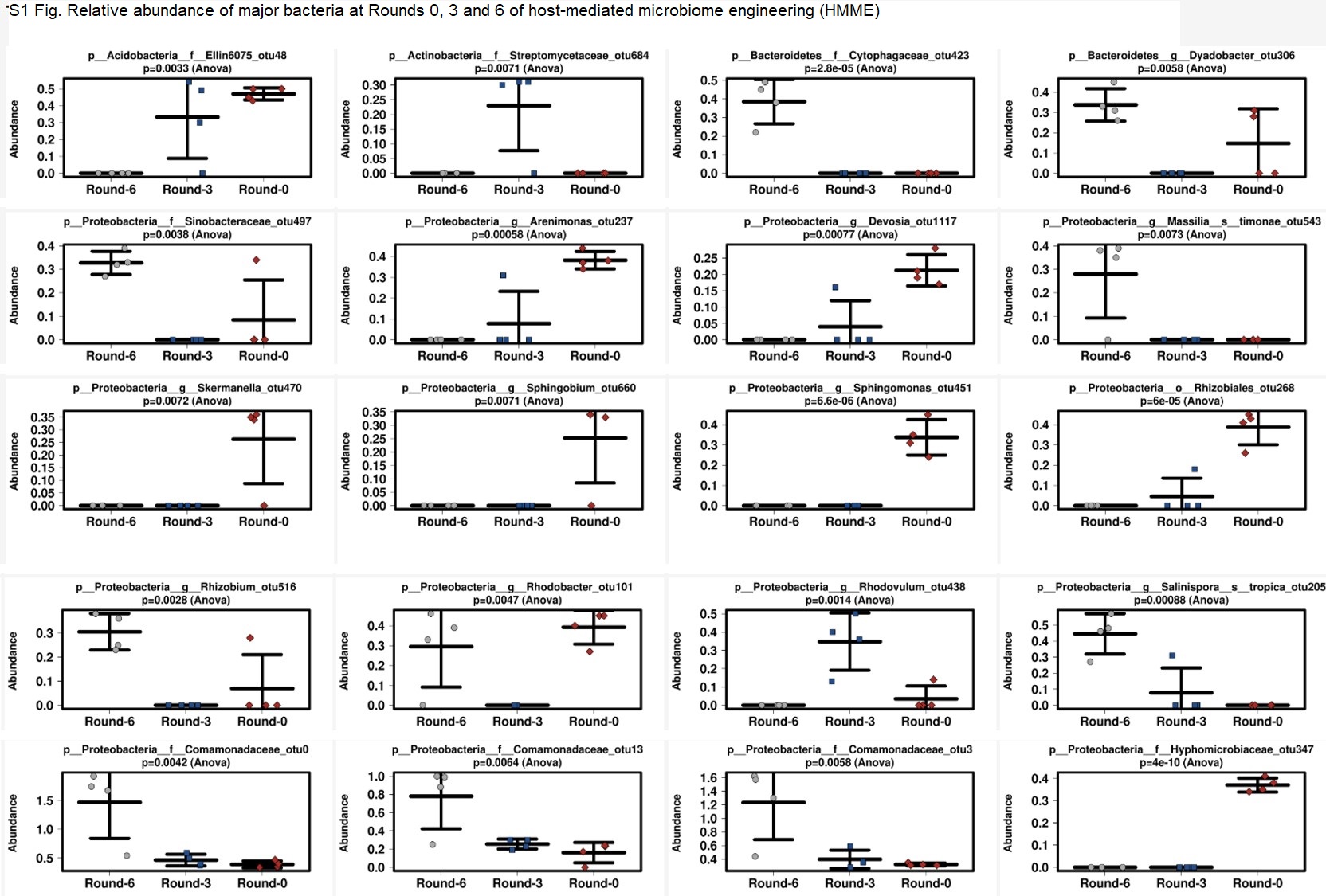

Supplement: S1 Fig — (JPG) [file pone.0225933.s002.jpg]
